# Implementation of disease activity measurement for rheumatoid arthritis patients in an academic rheumatology clinic

**DOI:** 10.1186/s12913-016-1633-x

**Published:** 2016-08-15

**Authors:** Alison Bays, Elizabeth Wahl, David I. Daikh, Jinoos Yazdany, Gabriela Schmajuk

**Affiliations:** 1 Division of Rheumatology, University of Washington, Harborview, 1959 NE Pacific Street, Box 356428, Seattle, WA USA; 2 Division of Rheumatology, Veterans Affairs Medical Center – San Francisco, 4150 Clement Street, Mailstop 111R, San Francisco, CA 94121 USA; 3 Division of Rheumatology, 1001 Potrero Ave, SFGH 30, San Francisco, CA 94110 USA

**Keywords:** Quality improvement, Rheumatoid arthritis, Disease activity

## Abstract

**Background:**

Treat-to-target is the recommended strategy for the management of rheumatoid arthritis (RA) and involves regular assessment of disease activity using validated measures and subsequent adjustment of medical therapy if patients are not in remission or low disease activity. Recommendations published in 2012 detailed the preferred disease activity measures but there have been few publications on implementation of disease activity measures in a real-world clinic setting.

**Methods:**

Plan-Do-Study-Act (PDSA) methodology was used over two cycles with a goal of increasing provider measurement of disease activity during all RA patient visits. In PDSA cycle 1, we implemented a paper-based form to help providers assess disease activity in RA patients. PDSA cycle 2 included the creation of separate patient and physician forms for collection of information, identification of patients prior to their clinic visit and incorporation of medical assistants into the workflow.

**Results:**

The first PDSA cycle improved the number of RA patients with documented disease activity measures from 24 % over a 4-week period, to an average of 44 % over an 8-week period. The second PDSA cycle showed a sustained and dramatic improvement, with 85 % of patients having a disease activity measure recorded over a 27-week period.

**Conclusions:**

Implementation of disease activity measurement in a typical academic rheumatology clinic can be achieved by standardizing workflow using a simple paper form.

**Electronic supplementary material:**

The online version of this article (doi:10.1186/s12913-016-1633-x) contains supplementary material, which is available to authorized users.

## Background

Current management of many chronic diseases, such as diabetes, hyperlipidemia and hypertension employ treatment strategies to achieve a specific quantitative clinical measure [1]. Recently, this “treat-to-target” approach has been applied to rheumatoid arthritis (RA) [2]. Instead of targeting a laboratory test or blood pressure measurement, the “target” in RA is disease activity, which can be measured using a variety of validated tools. Most of these combine swollen and tender joint counts, physician and patient global assessments and sometimes laboratory values [3]. A systematic review in 2010 assessing treat to target versus usual care strategies in RA revealed consistent improvement in clinical outcomes with treat to target: Patients managed with treat-to-target approaches had greater reductions in disease activity and a higher likelihood of achieving remission over usual care and additionally, experienced a reduction in radiographic erosions, improved physical function and quality of life [1, 4–6]. Current practice guidelines recommend patients with RA be treated with the goal of achieving remission or low disease activity [7].

In 2014, the National Quality Forum (NQF) endorsed measures requiring disease activity measurements in a majority of visits (greater than 50 %) in RA patients [8]. Acceptable measures of disease activity identified by the American College of Rheumatology include the Clinical Disease Activity Index (CDAI), Disease Activity Score with 28-joint counts (DAS28 ESR or CRP), Patient Activity Scales (PAS and PAS-II), Routine Assessment of Patient Index (RAPID-3) and Simplified Disease Activity Index (SDAI) [3].

Despite these recommendations for regular disease activity measurement in RA patients, routine use of these measurements has not been instituted in many rheumatology clinics. Reports from Physician Quality Reporting System (PQRS) indicate that in 2009, 630 of 83,849 physicians (<1 %), the majority of whom where rheumatologists, reported on one quality measure on RA patients. Among the physicians reporting one at least one measure for their RA patients (*n* = 630), they primarily reported disease-modifying antirheumatic drugs (DMARD) usage and only 29 % reported information on a disease activity measure (*n* = 183) [9].

Little has been published about how to best implement disease activity measurement in the clinic. Of the publications that discuss implementation, they are focused on the integration of software with their electronic health record [10, 11]. However, not all rheumatology clinics have the IT support or ability to develop or purchase advanced modules to modify their system.

In this study, we describe methods for implementation of disease activity measurement in a tertiary care rheumatology clinic. The San Francisco Veterans Affairs (VA) hospital, associated with University of California, San Francisco (UCSF) provided an ideal place to implement use of disease activity measures for patients with RA. Trainees working in the rheumatology clinic were familiar with the use of disease activity measures at other locations within the UCSF healthcare system. Baseline use of disease activity measurement in the clinic was infrequent prior to the start of the study, in part because the electronic medical record (EMR) does not provide an easy method for documenting joint scores. Modifying the SFVA’s EHR was not feasible in the short-term, so we planned an intervention using a paper scoring form. We assessed whether standardization of the clinic’s workflow to incorporate these forms increased the documentation of disease activity measures in the provider notes for patients with RA.

## Methods

### Study setting

The study took place in an academic rheumatology clinic at the VA Hospital in San Francisco. All providers working in the clinic were included in the intervention and analysis. The practice consists of nine rheumatology fellows (two fellows were present for only the pre-intervention period) one nurse practitioner and five attending physicians.

All patients with a diagnosis of rheumatoid arthritis in the medical record (reviewed by AB) were included. They study period lasted from June 2014 until April 2015. This project was considered exempt from IRB approval by the San Francisco VA IRB because it qualified as a quality improvement activity. Providers were aware of the interventions used. No funding sources were utilized for this quality improvement project.

### Preliminary work to identify workflow challenges

Preliminary work involved interviewing staff, fellows and faculty to understand the barriers to documentation of RA disease activity. Prior to the intervention, a clinician wishing to document a disease activity measure would have to 1) identify RA patients requiring a measure, 2) request this patient’s global assessment on a 0–10 or 0–100 Likert or visual analog scale during the clinical encounter 3) conduct the history and physical, including a 28-joint count, and 4) calculate the CDAI or DAS28 score and remember or look up the corresponding disease activity category 5) document a disease activity measure in the clinic note. No standard method existed for identifying patients ahead of clinic and providing them with a visual analog or Likert scale for rating their patient global assessment. Additionally, a ruler was often not available to score the patient and physician global if a visual analog scale was used. Other obstacles identified prior to the initiation of the first QI cycle were a lack of expectation on the part of the attending physicians to report a disease activity score, a lack of ability to modify the electronic medical record to incorporate the disease activity measures, and time constraints in clinic.

Upon reviewing the barriers to completing disease activity measures, the modifiable obstacles included attending physician expectations and availability of a form on which patients could indicate a global assessment and ruler with which to measure a visual analog scale line. The obstacles that were not immediately modifiable were changing the medical record (CPRS) and the time available to see a patient.

### Disease activity measures

The Clinical Disease Activity Index (CDAI) incorporates a patient global score on a 0 to 10 scale, a physician global score on a 0 to 10 scale and a 28-joint count for both tenderness and swelling. The score is tabulated with up to 28 points for tenderness, 28 points for swelling, 10 points for the physician global and 10 points for the patient global. These scores are then translated into four categories: remission, low disease activity, moderate disease activity or high disease activity [3].

The disease activity score 28 or DAS28 (ESR or CRP) incorporates the patient global score on a scale of 0–100, a tender joint count of 28 joints, a swollen joint count of 28 joints and either the erythrocyte sedimentation rate (ESR) or the C-reactive protein (CRP) and the provider then uses a formula to calculate the disease activity score and it is again characterized as remission, low disease activity, moderate disease activity or high disease activity [3].

### Interventions

A Plan-Do-Study-Act (PDSA) methodology provides a structure of iterative change that adapts to feedback to result in the desired outcome. The first stage, ‘plan’, is a planning stage in which a change is identified with the goal of improvement. The second stage, ‘do’, is when this change is tested. The third stage, ‘study’, examines the success of the change and the ‘act’ stage identifies the next steps needed for beginning the next PDSA cycle [12]. We undertook two PDSA cycles in an attempt to improve use and documentation of disease activity measures at the San Francisco VA rheumatology clinic. There was a 4-week pre-intervention period where we assessed use of disease activity measures prior to any specific intervention. A first PDSA cycle was planned and assessment of its effect on disease activity measurement documentation was planned for 8 weeks later. A second PDSA cycle was planned and assessment of its effect on disease activity measurement documentation was again planned for 8 weeks later. Because of the success of PSDA cycle 2, we continued to follow the outcome for an additional 21 weeks (about 5 months).

### Outcome

The outcome of interest was the proportion of RA patients seen on a given day in clinic who had a documented disease activity measure in the encounter note for that day. Eligible RA patients were identified prior to their clinic visit through medical chart review by one author (AB) based on the assessment sections on the most recent note. Presence or absence of the disease activity measure was determined by review of the entire note.

### Data analysis

We divided the study period into three parts – pre-intervention, PDSA cycle 1 and PDSA cycle 2. We created control charts to describe the proportion of encounters on each clinic day with a disease activity measure documented in the clinical note (p-charts). A continuous improvement of 6 or more points in the same direction is considered a trend [13].

## Results

Between week 1 and week 39, there were 107 RA patients seen at the SFVA rheumatology clinic. Basic patient characteristics are listed in Table 1. Of these 107 patients, the average age at their most recent appointment was 67 years. In the 103 patients whose rheumatoid factor (RF) and/or cyclic citrullinated peptide antibody (CCP) status were known, 81 (79 %) of these patients were seropositive (73 were CCP positive and 71 were RF positive), 38 patients (35 %) were on biologic agents (Table 1).

**Table 1:** Characteristics of rheumatoid arthritis patients seen in the San Francisco VA rheumatology clinic during study period

Characteristics	Rheumatoid arthritis patients
	*N* = 107
Age, mean (SD)	67 (10)
Male, N (%)	93 (87 %)
Seropositive,^a^ N (%) [*N* = 103]	81 (79 %)
Medications at end of study period, N (%)	
Methotrexate	60 (56 %)
Prednisone	42 (39 %)
Biologic	38 (35 %)

^a^Rheumatoid factor or anti-cyclic citrullinated peptide antibody positive at any point, among patients tested

### Pre-intervention

During the 4-week pre-intervention period, there were 29 RA patient encounters. Twenty-four percent of pre-intervention visits had a documented disease activity measure in the clinical note. A single clinician was responsible for nearly all of this disease activity score documentation.

### PDSA cycle 1

The cycle 1 intervention consisted of 1) developing a paper form that would be readily available in clinic for collecting patient and physician global assessments and tabulating joint counts using the homunculus (Additional file 1) and 2) Making the form available in clinic 3) Brief one-on-one educational discussions with incoming fellows on the use of this form, the calculation of CDAI and DAS 28, and the purpose and goal of disease activity measurement. The providers were encouraged to download a CDAI and DAS-28 calculator onto their smartphones [14].

**Fig. 1:**
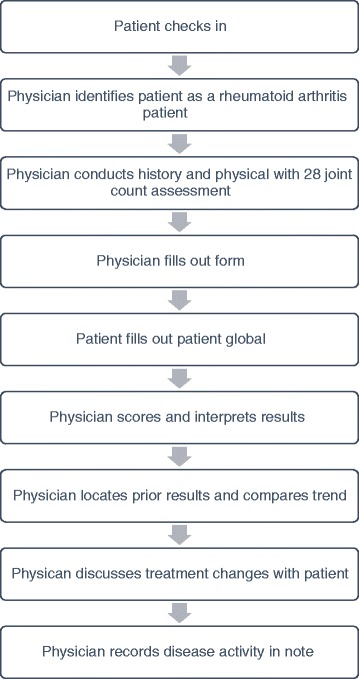
Cycle 1 workflow

In the workflow (Fig. 1) for cycle 1, providers were to identify RA patients and retrieve a form located in the clinic that included the patient global visual analog scale, physician global visual analog scale, a 28-joint count with homunculus and instructions on how to calculate the CDAI and DAS-28 ESR and CRP. The form included a fold-over ruler, obviating the need for finding a ruler, in addition to instructions for calculating the CDAI.

**Fig. 2:**
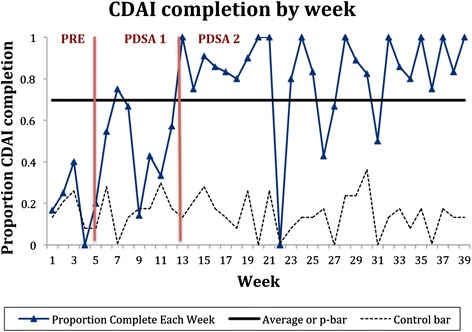
Percent completion of CDAI by week of intervention. The pre-intervention, PDSA Cycle 1, and PDSA Cycle 2 phases are separated by *red bars*. The *dark blue line* show sthe proportion of patient encounters with a documented CDAI by week. The upper control limits are not seen (>1). The lower control limits vary as the denominator of patient encounters changed each week. The p-bar shows the average CDAI documentation rate. More than 6 values are seen above the p-bar after Cycle 2 was implemented, indicating a positive improvement

PDSA cycle 1 lasted 8 weeks. During this period, providers had 59 visits with RA patients, and 26 (44 %) of those encounters had disease activity measures recorded in the note (see Fig. 2).

Follow-up interviews with clinicians identified several issues during cycle 1: 1) the patient and physician global were both completed on one form, so if the patient filled out the global score first, the physician would see the patient global score prior to the recording the physician global score, thus potentially biasing her rating; 2) the global assessment form had to be printed carefully so that the ruler lined up with the patient and physician global lines; 3) since RA patients were not pre-identified prior to their visits, providers would often forget to administer the patient global form.

### PDSA cycle 2

The cycle 2 intervention involved: 1) the creation of two separate forms so that the patients would complete the patient global in the waiting room, prior to the clinic visit, and the providers would not see the score until after completing their own global assessment; 2) additional education for providers; 3) identification and flagging of RA patients prior to the clinic visit so that patients could fill out global assessments in the waiting room prior to the start of their visit.

The one form used previously was split into two – the first form was designed for patients and included a global assessment scale (see Additional file 2). The second form was for the providers and included a 28-joint count homunculus to record the swollen and tender joints, a physician global score (see Additional file 3). It also included an explanation of how to calculate the CDAI and DAS-28. On both forms, the global scores were changed from a visual analog scale to a numerical Likert scale, noted to be interchangeable in American College of Rheumatology’s working group recommendations [3]. This eliminated the need for a ruler to calculate patient and provider global assessments.

Additional education was directed to providers. This included a post-clinic conference discussion of the history and utilization of disease activity measures and a division-wide Quality Improvement conference in which there was a discussion of the changes at the VA.

**Fig. 3:**
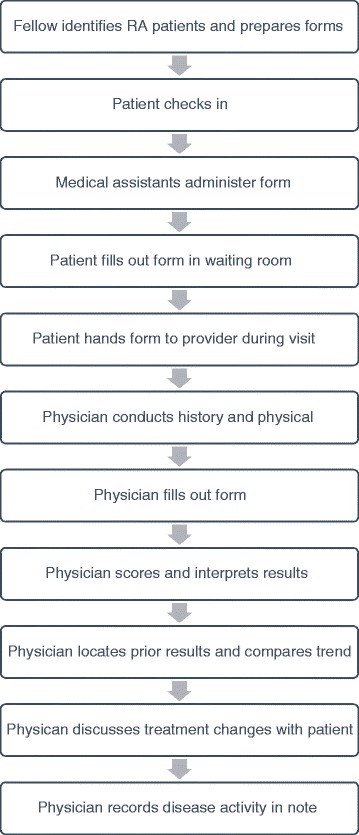
Cycle 2 workflow

RA patients were identified via chart review and flagged by a single author (AB) prior to clinic (see Fig. 3). Appropriate forms were provided to medical assistants prior to clinic. On the day of clinic, the medical assistants were instructed to give the form to flagged patients while taking their vital signs. The patients filled out the form while sitting in the waiting room. The patients would then either hand the form back to the medical assistant, who would attach it to the encounter form given to providers, or the patient could give the form directly to the clinician during the appointment. The physician would then conduct the history and physical, and enter the swollen and tender joint counts and the physician global into the table calculator on Form 2. The physician would then calculate the CDAI and/or DAS28 using simple addition or their IPhone app. When typing the note, the physician was instructed to include the score within the text of the note.

PDSA cycle 2 lasted 27 weeks. During this period, providers had 182 clinic visits with RA patients, and 155 (85 %) of those encounters had disease activity measures recorded in the note (see Fig. 2). Starting during PDSA cycle 2, there were six occurrences over the p-bar, which continued throughout the PDSA cycle, with three exceptions. All providers showed improvement in disease activity measure documentation. There were no outliers.

## Discussion

In this project, we describe the implementation of a reliable process for disease activity measurement for RA patients as recommended by the American College of Rheumatology in a single academic rheumatology clinic. Prior to the intervention, only 24 % of RA patients seen in clinic had documented disease activity measures and the majority of these patients had seen one third year fellow. By the end of the quality improvement initiative, we observed a sustained increase in the proportion of patients with of disease activity measures noted in the charts.

This project is generalizable to other clinics that may not have the capability to easily or immediately modify the electronic health system to incorporate disease activity measures. With minor changes to the workflow, we were able to incorporate the help of medical assistants, identify RA patients prior to their clinic appointment, and provide two forms for completion of global assessments, resulting in sustained improvement in reporting of disease activity measures. As disease activity measurement becomes a requirement of high quality care, we have shown that this “paper-and-pencil” intervention can result in successful documentation of disease activity measures without relying on changes to the electronic health record. This may be especially important for clinics with limited resources, or where changes to the health IT infrastructure may be difficult.

Our study has several limitations. First, it is difficult to disentangle which specific aspect of our intervention was the key improving disease activity measure documentation. Separating the effects of provider education or changes in culture from the effects of easy availability of patient global score for relevant patients is not possible given our study design. However, given the anemic improvement in the PDSA cycle 1, we can say that pre-clinic identification of patients and score collection in the waiting room seemed to be key attributes of a successful intervention. Second, our follow-up time is not long enough and our sample size not large enough to be able to witness changes in mean CDAI or DAS scores over time as a result of the implementation of this “treat to target” approach, although such improvements have been documented in trial settings previously [1, 4–6].

Future projects include exploring the possibility of modifying the electronic medical record to include the CDAI or DAS-28 as structured fields. This would allow for easy identification of patients with and without disease activity measures and allow clinicians to see trends in their performance over time. It would also make it easy to trend a single patient’s disease activity over time to evaluate their response to therapy. Modifying the electronic medical record so that it stores disease activity over time and allows providers to see trends over time is an ideal system that would assist providers in being able to assess trends over time. This has been implemented and has shown improvement in quality of care, efficiency of care and productivity [11]. However, this is not possible with all electronic medical records.

## Conclusions

Through utilization of a simple paper form and changes to workflow, rheumatology clinics can implement the use of standardized disease activity measures to offer patients high quality care consistent with the recommendations of the American College of Rheumatology [3, 15].

## Additional files

Additional file 1: Cycle 1 form (PDF 533 kb) Additional file 2: Cycle 2 patient form (PDF 137 kb) Additional file 3: Cycle 2 provider form (PDF 447 kb)

## Abbreviations

CCP: Cyclic Citrullinated Peptide Antibody
CDAI: Clinical Disease Activity Index
DAS28 ESR or CRP: Disease Activity Score with 28 Joint Counts
DMARD: Disease-Modifying Antirheumatic Drugs
EMR: Electronic Medical Record
NQF: National Quality Forum
PAS and PASII: Patient Activity Scales
PDSA: Plan Do Study Act
PQRS: Patient Quality Reporting System
RA: Rheumatoid Arthritis
RAPID-3: Routine Assessment of Patient Index
RF: Rheumatoid Factor
SDAI: Simplified Disease Activity Index
UCSF: University of California, San Francisco
VA: Veterans Affairs Hospital
