# Supplementary material for: Implementation of disease activity measurement for rheumatoid arthritis patients in an academic rheumatology clinic
Source: BMC Health Serv Res. 2016 Aug 15;16:384. doi: 10.1186/s12913-016-1633-x (PMC4986364; doi:10.1186/s12913-016-1633-x)
Supplement: Additional file 1: — Cycle 1 form (PDF 533 kb) [file 12913_2016_1633_MOESM1_ESM.pdf]

APPENDIX 1: PDSA Cycle 1 Form

PATIENT TO FILL OUT FRONT HALF

Considering all the ways that your arthritis affects you, rate how you are doing on the following scale by placing a vertical mark (|) on the line

Example:

0 —|———— 100

Very Well   Very Poor

0 ____ 100

PHYSICIAN TO FILL OUT

Physician global assessment of patient disease activity

Very Well   Very Poor

0 ____ 100

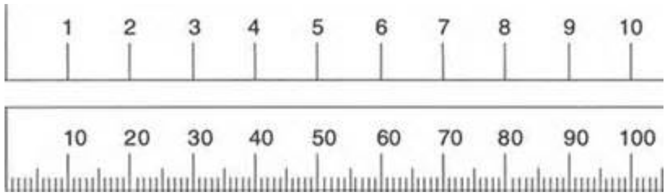

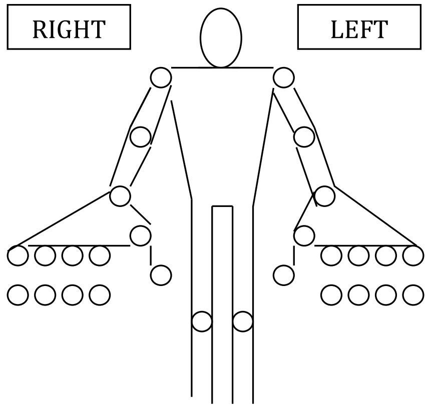

Notes:

Tender = 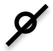 Swollen = 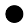 Tender & Swollen = 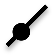

☐ No tender joints
☐ No swollen joints

CDAI: Tender and swollen joint counts (include MCPs, PIPs, wrists, elbows, shoulders and knees only), patient and provider global scores (this is calculated in cm), labs are not needed. Calculate below and put value into note.

How to Score the CDAI

**Variable**	**Range**	**Value**
Tender joint score	(0-28)	
Swollen joint score	(0-28)	
Patient global score	(0-10)	
Provider global score	(0-10)	
**Add the above values to calculate the CDAI score**	**(0-76)**	

**CDAI Score Interpretation**	
0.0 – 2.8	Remission
2.9 – 10.0	Low Activity
10.1 – 22.0	Moderate Activity
22.1 – 76.0	High Activity

DAS28: Tender and swollen joint counts (include MCPs, PIPs, wrists, elbows, shoulders and knees only), patient global score (this is calculated in mm), ESR or CRP. Use free app to calculate DAS28 (“DAS28 Calculator”) and put value into note.

Response Table		Changes in DAS 28 From Baseline		
		>1.2	0.6 – 1.2	< 0.6
Current Value DAS 28	< 3.2	good	moderate	poor
	3.2 – 5.1	moderate	moderate	poor
	> 5.1	moderate	poor	poor
