# Supplementary material for: Implementation of disease activity measurement for rheumatoid arthritis patients in an academic rheumatology clinic
Source: BMC Health Serv Res. 2016 Aug 15;16:384. doi: 10.1186/s12913-016-1633-x (PMC4986364; doi:10.1186/s12913-016-1633-x)
Supplement: Additional file 2: — Cycle 2 patient form (PDF 137 kb) [file 12913_2016_1633_MOESM2_ESM.pdf]

APPENDIX 2: Cycle 2 patient form

VA Patient Global Assessment

Patient Name: ____________________
Last four: _____________
Date: _________

Considering all of the ways your arthritis affects you, rate how well you are doing on the following scale:

o	o	o	o	o	o	o	o	o	o	o	o	o	o	o	o	o	o	o	o	o
0	0.5	1.0	1.5	2.0	2.5	3.0	3.5	4.0	4.5	5.0	5.5	6.0	6.5	7.0	7.5	8.0	8.5	9.0	9.5	10

Very Well Very Poor
