# Supplementary material for: Implementation of disease activity measurement for rheumatoid arthritis patients in an academic rheumatology clinic
Source: BMC Health Serv Res. 2016 Aug 15;16:384. doi: 10.1186/s12913-016-1633-x (PMC4986364; doi:10.1186/s12913-016-1633-x)
Supplement: Additional file 3: — Cycle 2 provider form (PDF 447 kb) [file 12913_2016_1633_MOESM3_ESM.pdf]

APPENDIX 3: Cycle 2 provider form

Patient Name: ______________________
Last 4: __________
Date: ____________

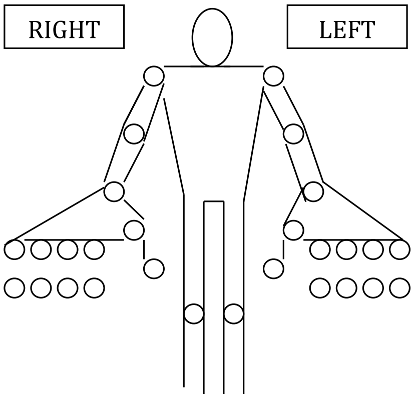

Tender = Swollen = Tender & Swollen =

☐ No tender joints
☐ No swollen joints

Provider Global Assessment of Disease Activity

Very Well	0	0.5	1.0	1.5	2.0	2.5	3.0	3.5	4.0	4.5	5.0	5.5	6.0	6.5	7.0	7.5	8.0	8.5	9.0	9.5	10	Very Poor

<u>CDAI</u>: Tender and swollen joint counts (include MCPs, PIPs, wrists, elbows, shoulders and knees only), patient and provider global scores (this is calculated in cm), labs are not needed. Calculate below and put value into note.

How to Score the CDAI

Variable	Range	Value
Tender joint score	(0-28)	
Swollen joint score	(0-28)	
Patient global score	(0-10)	
Provider global score	(0-10)	
**Add the above values to calculate the CDAI score**	**(0-76)**	

CDAI Score Interpretation	
0.0 – 2.8	Remission
2.9 – 10.0	Low Activity
10.1 – 22.0	Moderate Activity
22.1 – 76.0	High Activity

<u>DAS28</u>: Tender and swollen joint counts (include MCPs, PIPs, wrists, elbows, shoulders and knees only), patient global score (this is calculated in mm), ESR or CRP. Use free app to calculate DAS28 (“DAS28 Calculator”) and put value into note.

Response Table		Changes in DAS 28 From Baseline		
		>1.2	0.6 – 1.2	< 0.6
Current Value DAS 28	< 3.2	good	moderate	poor
	3.2 – 5.1	moderate	moderate	poor
	> 5.1	moderate	poor	poor
